# Pharmacogenomics of Hypertension and Preeclampsia: Focus on Gene–Gene Interactions

**DOI:** 10.3389/fphar.2018.00168

**Published:** 2018-02-28

**Authors:** Marcelo R. Luizon, Daniela A. Pereira, Valeria C. Sandrim

**Affiliations:** ^1^Department of General Biology, Institute of Biological Sciences, Federal University of Minas Gerais (UFMG), Belo Horizonte, Brazil; ^2^UFMG Graduate Program in Genetics, Institute of Biological Sciences, Federal University of Minas Gerais, Belo Horizonte, Brazil; ^3^Department of Pharmacology, Institute of Biosciences of Botucatu, Universidade Estadual Paulista, Botucatu, Brazil

**Keywords:** antihypertensive therapy, gene–gene interactions, epistasis, hypertension, pathways, pharmacogenomics, preeclampsia

## Abstract

Hypertension is a leading cause of cardiovascular mortality, but only about half of patients on antihypertensive therapy achieve blood pressure control. Preeclampsia is defined as pregnancy-induced hypertension and proteinuria, and is associated with increased maternal and perinatal mortality and morbidity. Similarly, a large number of patients with preeclampsia are non-responsive to antihypertensive therapy. Pharmacogenomics may help to guide the personalized treatment for non-responsive hypertensive patients. There is evidence for the association of genetic variants with variable response to the most commonly used antihypertensive drugs. However, further replication is needed to confirm these associations in different populations. The failure to replicate findings from single-locus association studies has prompted the search for novel statistical methods for data analysis, which are required to detect the complex effects from multiple genes to drug response phenotypes. Notably, gene–gene interaction analyses have been applied to pharmacogenetic studies, including antihypertensive drug response. In this perspective article, we present advances of considering the interactions among genetic polymorphisms of different candidate genes within pathways relevant to antihypertensive drug response, and we highlight recent findings related to gene–gene interactions on pharmacogenetics of hypertension and preeclampsia. Finally, we discuss the future directions that are needed to unravel additional genes and variants involved in the responsiveness to antihypertensive drugs.

## Introduction

Pharmacogenomics aims to elucidate the contribution of genetic variants to interindividual variability in drug responses, such as efficacy, dose requirements and adverse reactions. Candidate gene association studies have been performed to assess the effects of single polymorphisms or haplotypes, the combination of alleles at multiple polymorphisms, on drug response phenotypes. In the past decade, genome-wide association studies (GWAS) in pharmacogenomics have led to the identification of variants associated with efficacy and adverse drug reactions. However, much of the genetic heritability to drug response phenotypes appears to be hidden in multigenic and multifactorial complex traits (Zanger, 2010). It remains uncertain how many genes and genetic variants contribute to pharmacological traits, how common and rare variants affect response and whether gene–gene interactions play a role (Chhibber et al., 2014).

Gene–gene interactions among common regulatory variants can have strong effects in drug response phenotypes (Sadee et al., 2014). Notably, findings from 108 GWAS in pharmacogenomics revealed that 96.4% of the 928 associated single nucleotide polymorphisms (SNPs) are located in non-coding regions (Luizon and Ahituv, 2015). These findings suggest that most of common variants associated with variability in drug response are regulatory. However, most of the SNPs used in GWAS arrays were selected as informative genetic markers with higher allele frequencies and more likely to have greater power to detect association. In addition, the associated SNPs in GWAS are not necessarily the causal variant responsible for the phenotype, but may be in high linkage disequilibrium with the causal variant (Luizon and Ahituv, 2015). Notably, the failure to replicate findings from pharmacogenetic studies, particularly in the analysis of single-locus, has prompted the search for novel statistical methods to analyze the data. These methods take into account the biological complexity underlying drug response and examine potential epistatic interactions that may predict drug response, as reviewed elsewhere (Motsinger et al., 2007; Pander et al., 2010).

**FIGURE 1:**
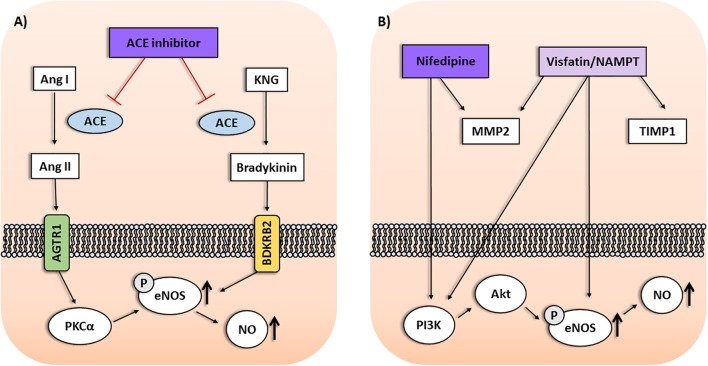
**(A)** Interactions among Protein Kinase C Alpha (*PRKCA*), *NOS3*, and *BDKRB2* gene products within the ACE inhibitor pathway may affect the antihypertensive response to enalapril. Protein kinase C alpha (PKCα) upregulates the endothelial nitric oxide synthase (eNOS) activity, leading to enhanced nitric oxide (NO) production and vasodilation. Moreover, stimulating bradykinin receptors on endothelial cells may result in eNOS upregulation mediated by PKCα. **(B)** Interactions among genes of the visfatin/NAMPT pathway and response to nifedipine in preeclampsia. *NAMPT* was associated with antihypertensive response in preeclampsia depending on *TIMP1* and *MMP2* genotypes. Nifedipine may affect circulating MMP levels in hypertension. Nifedipine and visfatin/NAMPT increase PI3K/Akt pathway and eNOS activity, resulting in enhanced NO production. Ang I, Angiotensin I; Ang II, Angiotensin II; AGTR1, Angiotensin II Receptor Type 1; KNG, Kininogen.

The multifactorial nature of drug response phenotypes require novel approaches to detect complex genetic effects from multiple minor impact genes, entire pathways, gene–gene and gene-environment interactions (Zanger, 2010). Therefore, research in pharmacogenomics should take into account the interactions among polymorphisms from different genes within drug response pathways. In this perspective article, we present current advances focused on gene–gene interactions associated with response to antihypertensive drugs, including non-coding SNPs found to be associated in GWAS and candidate gene studies, which are relevant to pharmacogenomics of hypertension and preeclampsia (**Figure 1**).

## Gene–Gene Interactions in Pharmacogenomics of Hypertension

Hypertension is the most prevalent modifiable risk factor for stroke and coronary heart disease, affects approximately 1 billion individuals worldwide and is a leading cause of cardiovascular mortality. GWAS have also improved the understanding on hypertension genetics, and provide evidence to suggest that much of the genetic influence on hypertension reside within non-coding elements and operate through gene–gene interactions (Padmanabhan and Joe, 2017). The polygenic nature of hypertension indicates that single loci may not be considered as relevant clinical target for all individuals (Padmanabhan and Joe, 2017). These findings highlight the importance of assessing the interactions among multiple loci when studying complex traits, including drug response phenotypes.

Antihypertensive therapy is associated with a significant reduction in mortality, and blood pressure (BP) is monitored in response to treatment and dosage adjusted on the basis of BP response and adverse effects. However, only about half of patients on antihypertensive therapy achieve BP control (Cooper-DeHoff and Johnson, 2016). Genetic variation may partially account for the interindividual variability in response to antihypertensive drugs. Candidate gene studies and GWAS have identified common genetic polymorphisms associated with response to antihypertensive drugs, as reviewed elsewhere (Fontana et al., 2015; Cooper-DeHoff and Johnson, 2016; Manunta et al., 2016). Although no examples have been sufficiently validated for clinical use, the available findings suggest promise and pharmacogenomics holds the potential to guide the personalized treatment for patients who are non-responsive to hypertensive drugs (Cooper-DeHoff and Johnson, 2016).

Hydrochlorothiazide is among the most commonly prescribed antihypertensive drugs. A GWAS found an intronic SNP (rs16960228, A/G) of the *PRKCA* gene encoding protein kinase C alpha (PKCα) to be associated with BP response to hydrochlorothiazide in two cohorts and replicated in other cohorts of European Americans (Turner et al., 2013). Systolic and diastolic BP responses were consistently greater in carriers of the GA+AA genotypes than in homozygote GG carriers across all study samples. The rs16960228 SNP was also associated with diastolic BP response to the β-blocker atenolol, with the opposite direction of effect, which may be explained by the different pharmacologic mechanisms compared to thiazide diuretics (Turner et al., 2013; Cooper-DeHoff and Johnson, 2016).

Angiotensin-converting enzyme inhibitors (ACEi) are also widely used to treat hypertension, including enalapril. The mechanism of action involves the reduction of angiotensin II formation, but a secondary mechanism is related to vasodilation produced by nitric oxide (NO) as a result of endothelial nitric oxide synthase (eNOS) activation. Both mechanisms seem to be affected by PKC signaling, as reviewed elsewhere (Oliveira-Paula et al., 2017) (**Figure 1A**). Recently, the rs16960228 SNP of *PRKCA* gene was also associated with BP responses in hypertensive patients classified as poor or good responders to enalapril. The GA+AA genotypes, and the A allele, were associated with worse responses to enalapril (Oliveira-Paula et al., 2017). Previous expression studies have shown that *PRKCA* expression was significantly higher among carriers of the GA+AA genotypes than among those with the GG genotype (Turner et al., 2013). It is possible that the GG genotype can enhance the responses to ACEi, which increase PKCα. Conversely, it seems reasonable to suggest that PKCα signaling could be less affected by ACEi in patients carrying the GA+AA genotypes, resulting in worse responses to these drugs. However, the molecular mechanisms supporting these suggestions remain to be proved (Oliveira-Paula et al., 2017).

Interactions among genes within the ACEi pathway might also influence the responses to enalapril. Polymorphisms of the bradykinin receptor B2 (*BDKRB2*) and nitric oxide synthase 3 (*NOS3*) genes were associated with the antihypertensive responses to enalapril (Silva et al., 2013; Oliveira-Paula et al., 2016). However, the CC genotype for the rs1799722 SNP (C-58T) in the promoter region of *BDKRB2* gene was not associated with the response to enalapril in single-locus analysis. Interestingly, the gene–gene interaction analysis showed that the CC genotype for the rs1799722 SNP was associated with response to enalapril depending on genotypes for the rs2070744 SNP (T-786C) in the promoter region of *NOS3* gene. The combination with the TT genotype was more frequent in poor responders, while the combination with TC genotype was more frequent in good responders (Silva et al., 2013). These findings are obscured when single *BDKRB2* genotypes alone are considered, and were found to be associated with drug response only when performing gene–gene interaction analysis.

Furthermore, interactions among *PRKCA*, *BDKRB2* and *NOS3* genes were also shown to affect the response to enalapril (Oliveira-Paula et al., 2017). In single-locus analysis, the GG genotype for the rs16960228 GWAS SNP of *PRKCA* gene was associated with better responses to enalapril. However, the combinations between *BDKRB2* and *NOS3* genes found to be associated with either poor responders or good responders to enalapril described above (Silva et al., 2013) were significant only when combined with the GG genotype for the rs16960228 SNP of *PRKCA* gene (Oliveira-Paula et al., 2017). PKCα increases the transcription of *NOS3* gene and upregulates eNOS activity, leading to enhanced NO production and vasodilation. Moreover, ACEi increases tissue bradykinin levels, stimulating bradykinin receptors on endothelial cells, which may result in *NOS3* upregulation mediated by PKCα, as reviewed elsewhere (Oliveira-Paula et al., 2017). These gene–gene interactions could affect eNOS activity and NO bioavailability (**Figure 1A**). Thus, to evaluate circulating nitrite levels (a marker of endogenous NO formation) and plasma eNOS activity may be a possible approach to clarify the underlying molecular mechanisms. In this regard, *NOS3* polymorphisms were shown to modify plasma and blood nitrite concentrations (Metzger et al., 2013; Oliveira-Paula et al., 2016). However, further studies are needed to examine the effects of gene interactions within this pathway on circulating nitrite levels and eNOS activity in patients treated with ACEi (Oliveira-Paula et al., 2017). Taken together, these findings highlight the relevance of take into consideration gene–gene interaction analysis in pharmacogenomics of hypertension.

## Gene–Gene Interactions in Pharmacogenomics of Preeclampsia

Preeclampsia is a major cause of maternal and perinatal morbidity and mortality, defined as pregnancy-induced hypertension and proteinuria after 20 weeks of gestation. It is a multisystem disorder that has implications for future maternal health, in particular an increased risk of cardiovascular disease. Preeclampsia is a complex genetic disorder, and it is probable that no single gene or variant will be identified as responsible for all cases of preeclampsia (Williams and Morgan, 2012). This scenario highlights the need to focus on more than one candidate gene, and on interactions among polymorphisms of different genes rather than on the effect of single polymorphisms. Indeed, interactions among SNPs in candidate genes within pathways relevant to disease pathophysiology were associated with the susceptibility to preeclampsia (Luizon et al., 2012, 2014, 2017b).

Antihypertensive therapy in preeclampsia includes methyldopa, nifedipine, hydralazine, and labetalol. These antihypertensive drugs allow the prolongation of gestation, thereby decreasing fetal and maternal adverse outcomes. However, a large subgroup of pregnant with preeclampsia is non-responsive to antihypertensive therapy and is associated with the worst clinical parameters (Luizon et al., 2017a). The use of pharmacogenomics may maximize the treatment outcome by reducing maternal and fetal morbidity and mortality associated with preeclampsia, as well as reducing adverse events associated with pharmacological therapy (Williams and Morgan, 2012). However, pharmacogenetics of antihypertensive therapy in preeclampsia was the scope of just a few studies, which examined the association of genetic polymorphisms with subgroups of patients who were responsive or non-responsive to antihypertensive therapy (Luizon et al., 2017a). Patients with preeclampsia were classified according to response to the initial drug of choice, methyldopa, and to the total antihypertensive therapy, including nifedipine and hydralazine, the latter used in cases of hypertensive crisis, according to the criteria of responsiveness reviewed elsewhere (Luizon et al., 2017a).

These pharmacogenetic studies included polymorphisms that modify the expression of candidate genes related to the pathophysiology of preeclampsia (Luizon et al., 2017a). Notably, endothelial dysfunction is associated with both hypertension and proteinuria. NO plays an important role in regulating endothelial function, and the eNOS enzyme is responsible for NO synthesis in the cardiovascular system. Reduced expression of eNOS consequently results in reduced NO bioavailability which plays a significant role in the endothelial dysfunction associated with preeclampsia (Sandrim et al., 2008). Therefore, eNOS is considered as a potential target for therapy for preeclampsia (Williams and Morgan, 2012). Calcium channel blockers (including nifedipine) may improve endothelial function and restore NO bioavailability, which could produce its effects by enhancing NO formation, thus counteracting the impaired NO bioavailability in preeclampsia (Sandrim et al., 2008) (**Figure 1B**). Nifedipine was shown to increase PI3K/Akt pathway and to increase eNOS activity and expression, as reviewed elsewhere (Luizon et al., 2016). Therefore, *NOS3* polymorphisms that modulate NO synthesis could also interfere with the response to nifedipine. Notably, polymorphisms and haplotypes of *NOS3* gene were associated with the disease (Muniz et al., 2012) or with the responsive and non-responsive subgroups of patients with preeclampsia (Sandrim et al., 2010).

Drugs that target the systemic vascular dysfunction were proposed as potential therapies for preeclampsia. Visfatin, an adipokine also known as nicotinamide phosphorybosil transferase (NAMPT), is considered a potential biomarker for vascular endothelial dysfunction. Notably, visfatin/NAMPT was shown to increase the expression and activity of eNOS in human endothelial cells, leading to an enhanced production of NO, as reviewed elsewhere (Peiro et al., 2010). Therefore, *NAMPT* polymorphisms that modulate visfatin/NAMPT levels could be evaluated as biomarkers for this pathway. Notably, the rs1319501 SNP (T-423C) in the promoter region and the rs3801266 SNP (A/G) in the intron 1 of *NAMPT* gene were shown to be associated with the susceptibility to preeclampsia (Luizon et al., 2015), and to affect plasma visfatin/NAMPT levels in patients who were non-responsive to total antihypertensive therapy (Luizon et al., 2017b). The TC+CC genotypes of the rs1319501 SNP and the AG+GG genotypes of the rs3801266 SNP were associated with lower and higher visfatin/NAMPT levels, respectively (Luizon et al., 2017b). However, further research on the interplay among visfatin/NAMPT levels, NO bioavailability and the response to antihypertensive therapy in preeclampsia are needed (Luizon et al., 2017b).

Visfatin/NAMPT induces endothelial cell proliferation and migration mediated by signaling pathways relevant to preeclampsia. It enhances the levels and activation of matrix metalloproteinases (MMP)-2/9 while decreasing the levels of tissue inhibitor of metalloproteinases (TIMP)-1/2 (Peiro et al., 2010). MMPs are endopeptidases that may interact with vasoactive peptides and contribute to the endothelial dysfunction in preeclampsia. Notably, the GG genotype, and the G allele, of the rs2070584 (T/G) SNP of *TIMP1* gene were associated with both the disease and with the patients with preeclampsia who were non-responsive to antihypertensive therapy (Luizon et al., 2014). Conversely, SNPs of *MMP2* and *NAMPT* genes were not found to be associated with the responsiveness to antihypertensive therapy in preeclampsia, as reviewed elsewhere (Luizon et al., 2017a). Interestingly, gene–gene interaction analysis showed that the GG genotype for the rs2070584 SNP of *TIMP1* was associated with preeclampsia and with responsiveness to total antihypertensive therapy in preeclampsia depending on genotypes for the rs3801266 SNP (A/G) of *NAMPT* gene. The combination with the AA was more frequent in the responsive patients, while the combination with the AG genotype was more frequent in the non-responsive patients (Luizon et al., 2017b). These combinations were significant only when combined with the CC genotype of the rs2285053 SNP (C-735C) of *MMP2* gene. These findings are obscured when single *MMP2* and *NAMPT* genotypes alone are considered, as they were not associated with the responsiveness in the single-locus analysis. Therefore, they further highlight the importance of gene–gene interactions in the genetics (Luizon et al., 2012) and pharmacogenetics of preeclampsia (Luizon et al., 2017a,b). Notably, calcium channel blockers (like nifedipine) were shown to affect circulating MMP levels in essential hypertensive patients, as reviewed elsewhere (Luizon et al., 2017a). However, further research into the molecular mechanisms underlying the interactions among *NAMPT*, *TIMP1* and *MMP2* and their relationship with the response to antihypertensive therapy in preeclampsia is needed (**Figure 1B**).

## Conclusion and Perspectives

Here, we present current findings related to gene–gene interactions on pharmacogenomics of hypertension (Silva et al., 2013; Oliveira-Paula et al., 2017) and preeclampsia (Luizon et al., 2014, 2017b). Of note, the reported association findings should be replicated in different populations. Moreover, the molecular mechanisms supporting these gene–gene interactions remain to be proved. Regarding preeclampsia, plasma from non-responsive and responsive patients was shown to evoke different gene expression profiles in human umbilical vein endothelial cells (HUVEC). Notably, nifedipine and hydralazine may act by upregulate or downregulate genes found to be downregulated or upregulated in HUVEC incubated with plasma from non-responsive patients (Luizon et al., 2016). The use of transcriptomics data holds promise for potential insights and implications to the pharmacogenomics of hypertension (Cooper-DeHoff and Johnson, 2016).

We expect that epigenomics research focused on the regulatory role of GWAS associated SNPs located in non-coding regions might provide a deeper understanding of pathways involved in the response to antihypertensive drugs. Notably, more than 96% of the associated SNPs from GWAS in pharmacogenomics reside in non-coding regions, which suggest these common variants are regulatory (Luizon and Ahituv, 2015). In addition, gene–gene interactions among common regulatory variants can have strong effects in drug response phenotypes (Sadee et al., 2014). Therefore, we further expect that gene–gene interaction analysis are more likely to be used for pharmacogenomics in the future.

Taken together, these efforts will guide further studies focused on gene–gene interactions within pathways relevant to antihypertensive drug response, which may enable the identification of novel pharmacogenomic biomarkers to guide personalized medicine for hypertension and preeclampsia.

## Author Contributions

MRL and DAP drafted the perspective article. All authors revised and approved it for publication.

## Conflict of Interest Statement

The authors declare that the research was conducted in the absence of any commercial or financial relationships that could be construed as a potential conflict of interest.
